# Self-reported ADHD symptoms among pharmacy students: prevalence of positive ADHD symptom screening, academic correlates, and implications for educational support

**DOI:** 10.3389/fpsyt.2026.1872812

**Published:** 2026-07-23

**Authors:** Malaz J. Gazzaz, Mohammed M. Aldurdunji, Mohammad A. Alhazmi, Mohammed S. Alsubhi, Turky B. Bahhah, Ashraf N. Abdalla, Roaya S. Alqurashi, Alaa A. Khojah, Saad M. Wali

**Affiliations:** 1 Pharmaceutical Practices Department, College of Pharmacy, Umm Al-Qura University, Makkah, Saudi Arabia; 2 College of Pharmacy, Umm Al-Qura University, Makkah, Saudi Arabia; 3 Pharmacology and Toxicology Department, College of Pharmacy, Umm Al-Qura University, Makkah, Saudi Arabia; 4 Pharmaceutical Sciences Department, College of Pharmacy, Umm Al-Qura University, Makkah, Saudi Arabia

**Keywords:** ADHD, adult ADHD self-report scale, mental health, methylphenidate, pharmacy students, prevalence, Saudi Arabia

## Abstract

**Introduction:**

Attention-deficit/hyperactivity disorder (ADHD) is a prevalent neurodevelopmental condition that persists into adulthood and significantly impacts academic performance and quality of life. Despite growing recognition of adult ADHD globally, data on ADHD prevalence among pharmacy students in Saudi Arabia specifically remain scarce or not well characterized. This study aimed to estimate the prevalence of positive ADHD symptom screening based on self-reported symptoms consistent with ADHD among pharmacy students at Umm Al-Qura University, examine associated demographic and academic factors, and assess students’ knowledge of ADHD pharmacotherapy.

**Methods:**

A cross-sectional, questionnaire-based study was conducted among undergraduate pharmacy students at Umm Al-Qura University, Makkah, Saudi Arabia, between November and December 2025. A total of 203 students participated, corresponding to a response rate of 18.2%. The Adult ADHD Self-Report Scale (ASRS) version 1.1 Screener was used to screen for symptoms consistent with ADHD. Descriptive statistics, chi-squared tests, and multivariable logistic regression were employed for data analysis.

**Results:**

A total of 203 pharmacy students participated in the study (61.6% male, 38.4% female). The prevalence of positive ADHD symptom screening based on the ASRS version 1.1 Screener was 31.0% (n=63). Only 5.9% (n=12) reported having a formal ADHD diagnosis, while 29.1% (n=59) reported a personal belief that they might have undiagnosed ADHD (a subjective perception, distinct from screening or diagnostic criteria). No significant associations were found between ADHD screening status and gender, year of study, GPA, or daily study hours. However, students with higher GPA (>3.0) were significantly more likely to correctly identify methylphenidate as first-line ADHD treatment (OR = 2.57, 95% CI: 1.22-5.34, p = 0.012).

**Conclusion:**

A substantial proportion of pharmacy students screened positive for symptoms consistent with ADHD, yet the rate of self-reported prior diagnosis was low. These findings support the need for further evaluation of awareness and screening approaches in pharmacy education settings.

## 1 Introduction

Attention-deficit/hyperactivity disorder (ADHD) is a prevalent neurodevelopmental condition characterized by persistent inattention, hyperactivity, and impulsivity (1). Contemporary evidence demonstrates that ADHD persists in adulthood in approximately 50-65% of cases, with a worldwide prevalence estimated at 2.5-3.4% based on comprehensive meta-analyses (2–4). The condition can pose substantial challenges to academic achievement and occupational functioning, underlining its relevance in higher-education settings (5, 6).

University students represent a population of particular interest, as academic demands can unmask symptoms previously managed or unrecognized (7). Studies report elevated ADHD prevalence among university students, ranging from 2% to 12% worldwide (8, 9). Healthcare students face additional challenges given intensive curricula and high-stakes clinical responsibilities.

In Saudi Arabia, emerging research has characterized ADHD prevalence among medical students, with rates ranging from 10.9% to 26% across different regions (10, 11). A study at King Abdulaziz University found that 11.9% of undergraduates met criteria for suspected adult ADHD, with notably low prior diagnosis rates (6.5%), suggesting substantial under-recognition (10).

Despite this growing literature, data on ADHD prevalence among pharmacy students in Saudi Arabia remain scarce or not well characterized. This knowledge gap is particularly important given the demanding nature of pharmacy education, which can affect students’ well-being and academic performance. ADHD symptoms may further impair students’ ability to manage these challenges, underscoring the importance of assessing their prevalence and awareness to better understand their impact on quality of life and academic success.

Given these academic and professional demands, it is also important for pharmacy students to recognize potential ADHD symptoms to seek appropriate support and manage their condition effectively. In addition, pharmacy students will become healthcare providers responsible for dispensing controlled substances such as methylphenidate; their understanding of ADHD may influence the quality of care provided. Pharmacists are also often the last healthcare professionals patients interact with before initiating treatment, placing them in a unique position to identify adherence issues and adverse effects. Furthermore, the pharmacy curriculum requires sustained attention for tasks such as dosage calculations and drug interaction verification—demands that may be particularly challenging for students with unrecognized ADHD.

A Thai study reported that 14.4% of pharmacy students exhibited ADHD-like symptoms (12), but no comparable data from the Middle East are currently available. Pharmacists play an increasingly important role in ADHD management through patient education and medication optimization (13), making their awareness of ADHD particularly relevant.

This study aimed to investigate the prevalence of positive ADHD symptom screening among pharmacy students at Umm Al-Qura University using the Adult ADHD Self-Report Scale (ASRS) version 1.1 Screener. Specifically, the study sought to estimate the prevalence of symptoms consistent with ADHD based on screening results, examine associations with demographic and academic characteristics, assess the proportion of students with a formal ADHD diagnosis compared with those who self-perceived having ADHD, and evaluate knowledge of first-line ADHD treatment.

## 2 Methods

### 2.1 Study design

This was a cross-sectional, questionnaire-based study conducted among undergraduate pharmacy students at Umm Al-Qura University in Makkah, Saudi Arabia. Data collection occurred between November and December 2025.

### 2.2 Participants and setting

The study population consisted of undergraduate students enrolled in the Doctor of Pharmacy (Pharm.D) program at Umm Al-Qura University. All academic years were eligible for participation. Inclusion criteria were enrollment as an undergraduate student in the Pharm.D program at Umm Al-Qura University during the study period. No specific exclusion criteria were applied beyond non-enrollment in the program. A convenience sampling approach was employed, with questionnaires distributed electronically to all registered pharmacy students across all academic years. Participation was anonymous and voluntary, which is particularly important given the sensitivity of ADHD symptom disclosure. To maximize participation, reminder messages were sent periodically during the data collection period.

### 2.3 Survey instrument

The survey instrument consisted of multiple sections designed to comprehensively assess ADHD symptoms, demographic characteristics, and ADHD-related knowledge. The first section collected demographic and academic information including gender, year of study, current GPA, and average daily study hours. All demographic and academic variables were self-reported by participants. Although participants’ age was collected, it was not included in the analysis because the study population consisted of undergraduate Pharm.D students with a relatively homogeneous age distribution, and academic year was considered a more informative educational variable for the analyses performed. The second section incorporated the Adult ADHD Self-Report Scale (ASRS) version 1.1 Screener, a six-item screening instrument developed by the World Health Organization in conjunction with Kessler and colleagues (14). The questionnaire, including the ASRS version 1.1 Screener, was administered in English, as English is the official language of instruction in the Doctor of Pharmacy (Pharm.D) program at Umm Al-Qura University and is routinely used in students’ coursework and assessments. The ASRS version 1.1 Screener assesses the frequency of core ADHD symptoms over the preceding six months using a 5-point Likert scale ranging from “Never” (0) to “Very Often” (4). The six items evaluate symptoms including difficulty completing final details of tasks, difficulty organizing tasks, problems remembering appointments, fidgeting, and feelings of being overly active. The ASRS has demonstrated strong psychometric properties with high internal consistency (Cronbach’s alpha = 0.88), high sensitivity, and good specificity in adult populations (15–17). The present study used the ASRS version 1.1 Screener based on DSM-IV criteria. Additional sections assessed history of formal ADHD diagnosis, current ADHD medication use, self-perceived beliefs about having ADHD, and knowledge of ADHD pharmacotherapy including identification of first-line treatments and common side effects. The knowledge items were developed by the research team based on the existing literature and reviewed for content and clarity, then pilot-tested with a small group of students before distribution.

### 2.4 Scoring and categorization of ASRS

The Adult ADHD Self-Report Scale (ASRS) version 1.1 Screener consists of six items assessing core ADHD symptoms, each rated on a 5-point Likert scale ranging from “Never” (0) to “Very often” (4), yielding a total possible score from 0 to 24. Participants were categorized as having symptoms highly consistent with ADHD when four or more responses met the predefined frequency thresholds specified in the ASRS scoring guidelines, indicating a positive screening result and the need for further clinical assessment (14).

### 2.5 Ethical considerations

Ethical approval for this study was obtained from the Scientific Biomedical Research Ethics Committee, College of Medicine, Umm Al-Qura University (Approval No. HAPO-02-K-012-2025-02-2536). The study was conducted in accordance with the principles of the Declaration of Helsinki. Electronic informed consent was obtained from all participants prior to participation.

### 2.6 Statistical analysis

Statistical analyses were performed using RStudio (version 2024.9.1.394) with R (version 4.4.2). Descriptive statistics were calculated for all variables, with categorical variables presented as frequencies and percentages and continuous variables as means with standard deviations or medians with interquartile ranges as appropriate. Associations between ADHD screening status and categorical demographic and academic variables were examined using chi-squared tests or Fisher’s exact tests where expected cell counts were less than five. Multivariable logistic regression was employed to identify independent predictors of ADHD screening status and treatment knowledge, with results expressed as odds ratios (OR) with 95% confidence intervals (CI). A p-value of less than 0.05 was considered statistically significant for all analyses.

## 3 Results

**Figure 1:**
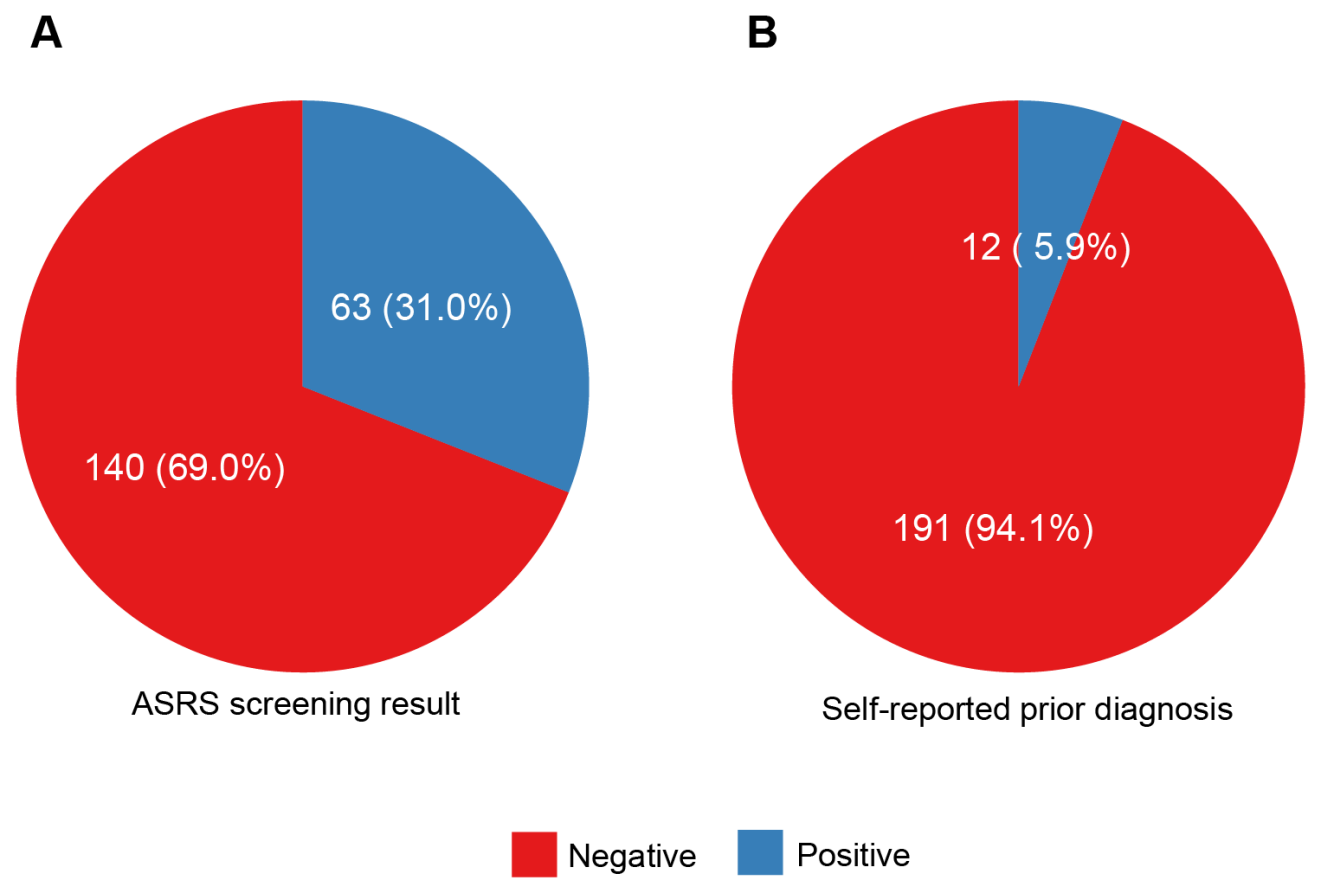
Frequencies and proportions of positive ADHD symptom screening based on the adult ADHD self-report scale (ASRS) version 1.1 screener **(A)** and self-reported prior formal ADHD diagnosis **(B)**.

**Figure 2:**
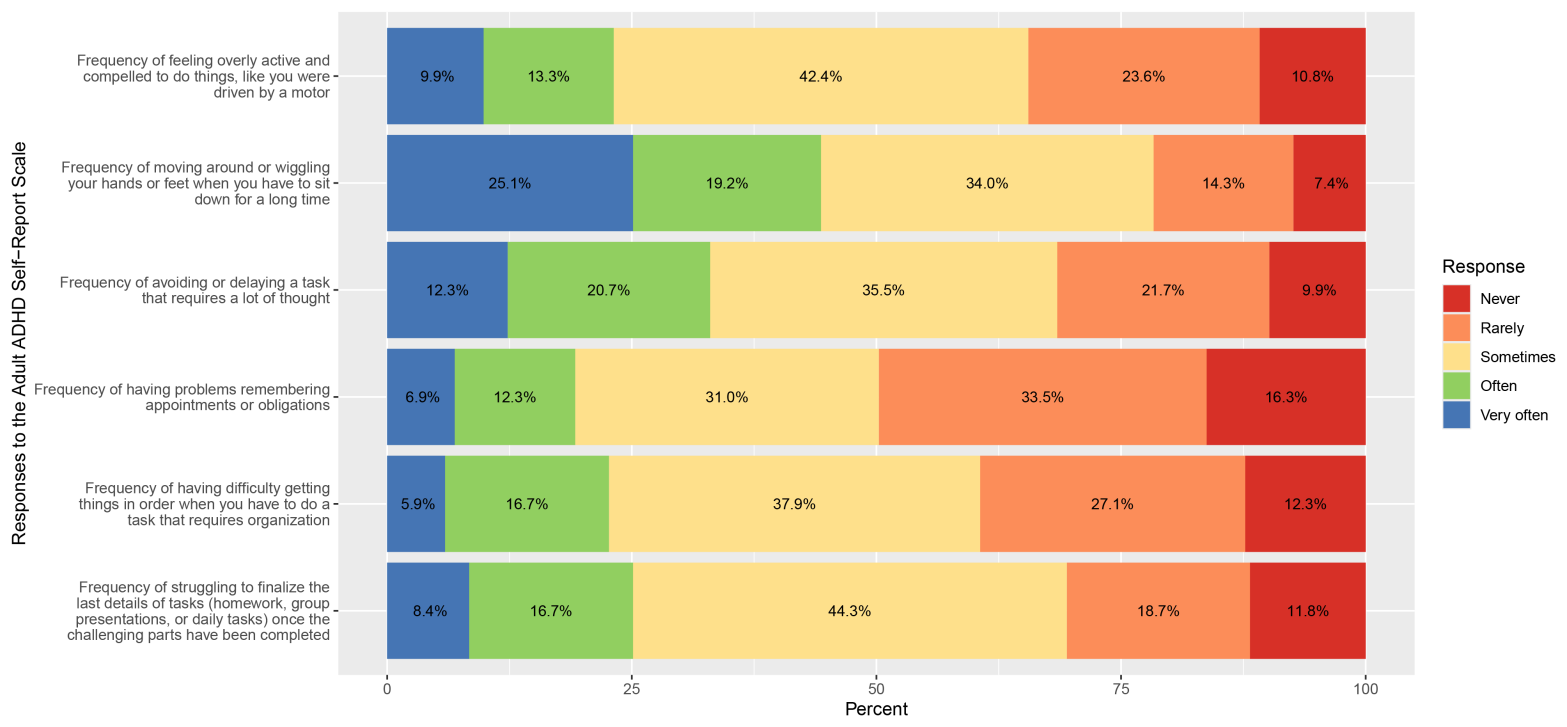
Distribution of participants’ responses to the six items of the adult ADHD self-report scale (ASRS) version 1.1 screener.

A total of 203 pharmacy students completed the survey. Detailed demographic characteristics, ASRS scores, ADHD diagnosis history, and treatment knowledge are presented in **Tables 1**–**5**, **Figures 1**, **2**. Among the participants, 61.6% (n=125) were male and 38.4% (n=78) were female. Intermediate (3rd + 4th year) students comprised the largest proportion (38.4%, n=78), followed by Junior (1st + 2nd year) students (32.0%, n=65) and Senior (5th + 6th year) students (29.6%, n=60). The majority of students (76.8%, n=156) reported a GPA between 3.1 and 4.0, while 19.2% (n=39) had a GPA between 2.1 and 3.0, and 3.9% (n=8) had a GPA ≤ 2.0. Regarding average daily study time, 16.3% (n=33) reported studying for less than 1 hour per day, 27.1% (n=55) for 1–2 hours, 34.5% (n=70) for 3–4 hours, 11.8% (n=24) for 5–6 hours, and 10.3% (n=21) for more than 6 hours per day. Based on a total enrolled pharmacy student population of 1,118 during the study period, this corresponds to a response rate of 18.2%.

**Table 1:** Demographic and academic characteristics.

Characteristic	Description
Gender	
Male	125 (61.6%)
Female	78 (38.4%)
Year of study	
Junior (1st + 2nd year)	65 (32.0%)
Intermediate (3rd + 4th year)	78 (38.4%)
Senior (5th + 6th year)	60 (29.6%)
Current GPA (on a scale of 0 to 4.0)	
≤2.0	8 (3.9%)
2.1 to 3	39 (19.2%)
3.1 to 4.0	156 (76.8%)
Average hours spent studying each day	
<1 hour	33 (16.3%)
1–2 hours	55 (27.1%)
3–4 hours	70 (34.5%)
5–6 hours	24 (11.8%)
More than 6 hours	21 (10.3%)

n (%).

**Table 2:** Factors and predictors of having symptoms highly consistent with ADHD based on the ASRS scale.

Characteristic	Positive ADHD screening			Multivariable regression		
	No N=140	Yes N=63	p-value	OR	95% CI	p-value
Gender			0.053			
Male	80 (64.0%)	45 (36.0%)		Reference	Reference	
Female	60 (76.9%)	18 (23.1%)		0.69	0.29, 1.61	0.389
Year of study			0.109			
Junior	51 (78.5%)	14 (21.5%)		Reference	Reference	
Intermediate	52 (66.7%)	26 (33.3%)		1.53	0.64, 3.69	0.340
Senior	37 (61.7%)	23 (38.3%)		1.79	0.69, 4.77	0.237
Current GPA			0.220			
≤3	29 (61.7%)	18 (38.3%)		Reference	Reference	
3.1 to 4.0	111 (71.2%)	45 (28.8%)		0.62	0.31, 1.26	0.183
Average hours spent studying each day			0.664			
<3 hours	58 (65.9%)	30 (34.1%)		Reference	Reference	
3–4 hours	49 (70.0%)	21 (30.0%)		1.00	0.49, 2.04	0.995
>4 hours	33 (73.3%)	12 (26.7%)		1.03	0.41, 2.55	0.942

n (%).

Pearson’s Chi-squared test.

CI, Confidence Interval; OR, Odds Ratio.

Results of multivariable logistic regression model assumptions: model fit (Hosmer–Lemeshow p = 0.674), McFadden’s R² = 0.028, multicollinearity (no variables had VIF > 5), and influential observations (no observations had Cook’s distance > 1). All variables were entered simultaneously.

**Table 3:** Characteristics of ADHD diagnosis, beliefs and perceptions.

Characteristic	Description
Ever diagnosed with ADHD	
No	191 (94.1%)
Yes	12 (5.9%)
If yes, currently taking medication for ADHD	
No	5 (41.7%)
Yes	7 (58.3%)
Believe that you have ADHD	
No	144 (70.9%)
Yes	59 (29.1%)
First-line treatment for ADHD	
Methylphenidate (Ritalin, Concerta)	157 (77.3%)
Donepezil (Aricept)	15 (7.4%)
Levodopa/Carbidopa (Sinemet)	13 (6.4%)
Carbamazepine (Tegretol)	18 (8.9%)
Known side effects of methylphenidate*	
Decreased appetite	146 (71.9%)
Dry mouth	88 (43.3%)
Headache	157 (77.3%)
Insomnia	159 (78.3%)
Urinary retention	59 (29.1%)

*An asterisk indicates a multiple-response item.

n (%).

**Table 4:** Factors and predictors of having a confirmed diagnosis of ADHD.

Characteristic	Confirmed diagnosis of ADHD		
	No N=191	Yes N=12	p-value
Gender			0.378
Male	116 (92.8%)	9 (7.2%)	
Female	75 (96.2%)	3 (3.8%)	
Year of study			0.933
Junior	61 (93.8%)	4 (6.2%)	
Intermediate	74 (94.9%)	4 (5.1%)	
Senior	56 (93.3%)	4 (6.7%)	
Current GPA			>0.999
≤3	44 (93.6%)	3 (6.4%)	
3.1 to 4.0	147 (94.2%)	9 (5.8%)	
Average hours spent studying each day			0.742
<3 hours	84 (95.5%)	4 (4.5%)	
3–4 hours	65 (92.9%)	5 (7.1%)	
>4 hours	42 (93.3%)	3 (6.7%)	

n (%).

Fisher’s exact test.

**Table 5:** Factors and predictors of providing correct responses regarding the first-line treatment for ADHD.

Characteristic	First-line treatment for ADHD*			Multivariable regression		
	Incorrect N=46	Correct N=157	p-value	OR	95% CI	p-value
Gender			0.205			
Male	32 (25.6%)	93 (74.4%)		Reference	Reference	
Female	14 (17.9%)	64 (82.1%)		2.01	0.77, 5.47	0.161
Year of study			0.795			
Junior	13 (20.0%)	52 (80.0%)		Reference	Reference	
Intermediate	18 (23.1%)	60 (76.9%)		1.04	0.40, 2.73	0.930
Senior	15 (25.0%)	45 (75.0%)		1.04	0.36, 3.00	0.948
Current GPA			0.012			
≤3	17 (36.2%)	30 (63.8%)		Reference	Reference	
3.1 to 4.0	29 (18.6%)	127 (81.4%)		2.57	1.22, 5.34	0.012
Average hours spent studying each day			0.386			
<3 hours	22 (25.0%)	66 (75.0%)		Reference	Reference	
3–4 hours	12 (17.1%)	58 (82.9%)		1.31	0.58, 3.05	0.523
>4 hours	12 (26.7%)	33 (73.3%)		0.62	0.23, 1.64	0.333

n (%).

Pearson’s Chi-squared test.

CI, Confidence Interval; OR, Odds Ratio.

*Incorrect responses included Donepezil, Levodopa/Carbidopa, or Carbamazepine, whereas Methylphenidate was the correct answer.

Results of multivariable logistic regression model assumptions: model fit (Hosmer–Lemeshow p = 0.741), McFadden’s R² = 0.047, multicollinearity (no variables had VIF > 5), and influential observations (no observations had Cook’s distance > 1). All variables were entered simultaneously.

Based on the ASRS version 1.1 Screener, 31.0% (n=63) of participants screened positive for ADHD symptoms. The internal consistency of the ASRS in this sample was acceptable (Cronbach’s alpha = 0.731). The median total ASRS score was 12.0 (IQR: 10.0-14.0), with individual item scores ranging from a median of 1.0 to 3.0. Regarding diagnostic history, only 5.9% (n=12) of participants reported having a formal ADHD diagnosis, while 29.1% (n=59) believed they had ADHD without formal diagnosis. Among those with formal diagnoses, 58.3% (n=7) reported currently taking ADHD medication.

No statistically significant associations were found between positive ADHD symptom screening and gender (p = 0.053), year of study (p = 0.109), current GPA (p = 0.220), or average daily study hours (p = 0.664). Multivariable logistic regression likewise identified no significant independent predictors of positive ADHD symptom screening.

Regarding ADHD treatment knowledge, 77.3% (n=157) of students correctly identified methylphenidate as a commonly recommended first-line pharmacological treatment for ADHD. To assess the predictors of basic knowledge of ADHD pharmacotherapy, a single binary knowledge item was included, where responses were coded as binary (correct identification of methylphenidate vs. incorrect) and analyzed without formal validity testing, as this represented a single knowledge question rather than a comprehensive assessment tool. Multivariable logistic regression analysis revealed that students with higher GPA (>3.0) were significantly more likely to correctly identify methylphenidate as a commonly recommended first-line pharmacological treatment for ADHD compared to those with lower GPA (OR = 2.57, 95% CI: 1.22-5.34, p = 0.012). Common side effects most frequently identified by students included insomnia (78.3%, n=159), headache (77.3%, n=157), and decreased appetite (71.9%, n=146).

## 4 Discussion

This study investigated the prevalence of positive ADHD symptom screening among pharmacy students at Umm Al-Qura University in Saudi Arabia, revealing that nearly one-third (31.0%) of participants screened positive for symptoms highly consistent with ADHD based on the ASRS version 1.1 Screener. Despite this high screening-positive proportion, only 5.9% reported having a formal ADHD diagnosis, suggesting a possible gap between symptom screening and formal diagnosis in this population. Additionally, 29.1% of students believed they had ADHD without formal diagnosis, and pharmacy students demonstrated generally good knowledge of ADHD pharmacotherapy, with 77.3% correctly identifying methylphenidate as first-line treatment. It is important to note that a positive ASRS screen indicates symptoms consistent with ADHD rather than a confirmed diagnosis; recent evidence suggests that self-report screening tools alone have limited accuracy for diagnosing ADHD (18).

The 31% prevalence of positive ADHD symptom screening observed in our study is notably higher than general population estimates of adult ADHD, which typically range from 2.5% to 3.4% globally (2–4). This higher proportion of positive ADHD symptom screening is consistent with patterns observed in other university student populations, where positive ADHD symptom screening has been reported at higher levels than in the general adult population (7–9). Our findings align with recent Saudi Arabian studies that have reported similarly elevated rates among healthcare students, ranging from 10.9% to 26% (10, 11). The prevalence in our pharmacy student sample is higher than the range reported for healthcare student populations.

Interestingly, a recent study from Thailand found that 14.4% of pharmacy students exhibited ADHD-like symptoms (12), which is lower than our observed rate, potentially suggesting regional or cultural differences in symptom reporting or positive ADHD symptom screening.

The discrepancy between the high proportion of positive ADHD symptom screening (31%) and the low rate of formal diagnosis (5.9%) observed in our study is particularly concerning. A similar pattern has been reported in other studies conducted in Saudi Arabia, where only 6.5% of students with suspected ADHD had received a formal diagnosis during childhood (10). This substantial diagnostic gap may reflect multiple factors, including limited awareness of ADHD as an adult condition in the region, insufficient mental health screening practices, cultural stigma associated with mental health diagnoses, or limited access to specialized diagnostic services (19). Supporting this interpretation, a recent retrospective study from the United Arab Emirates reported that adult ADHD diagnoses occurred predominantly within psychiatric services and were frequently accompanied by psychiatric comorbidities, highlighting the clinical complexity of adult ADHD presentations and the potential for under-recognition outside specialist mental health settings (20). The finding that 29.1% of students reported believing they might have undiagnosed ADHD may indicate a perceived gap between self-recognized symptoms and formal clinical evaluation. However, this high self-perceived belief may not solely indicate clinical under-recognition; it could also be influenced by the widespread availability of non-validated information on social media platforms, which may lead students to misattribute common academic burnout, stress, or temporary focus difficulties to adult ADHD.

Notably, our study found no statistically significant associations between ADHD screening status and any of the examined demographic or academic variables, including gender, year of study, GPA, or daily study hours. The lack of gender differences (p = 0.053) contrasts with some international literature suggesting higher ADHD prevalence in males (19), though this finding approaches statistical significance and may reflect the relatively balanced gender distribution in our sample or potential differences in symptom presentation and recognition between genders. The absence of significant associations with academic performance variables is somewhat surprising, as ADHD has been consistently linked to academic difficulties in the literature (5, 9, 21). However, this may reflect the highly selected nature of pharmacy students who have succeeded academically despite potential ADHD symptoms, suggesting effective compensatory strategies or self-selection out of higher education by more severely affected individuals.

A particularly relevant finding for pharmacy education is that 77.3% of students correctly identified methylphenidate as the first-line treatment for ADHD, with higher academic performers demonstrating significantly greater knowledge (OR = 2.57, 95% CI: 1.22-5.34, p = 0.012). This level of pharmacotherapy knowledge is encouraging, as future pharmacists will be responsible for dispensing ADHD medications and providing patient counseling. Notably, pharmacists occupy a unique position in ADHD care: they are often the most accessible healthcare providers, frequently interact with patients at each prescription refill, and are ideally placed to monitor adherence, identify side effects, and address medication concerns. For pharmacy students who may themselves experience ADHD symptoms, this dual perspective — as both future healthcare providers and potentially affected individuals — could enhance their empathy and counseling effectiveness with ADHD patients. Current clinical guidelines from organizations including the American Academy of Pediatrics and the National Institute for Health and Care Excellence (NICE) recommend stimulant medications, particularly methylphenidate, as first-line pharmacological treatment for ADHD (22, 23). This recommendation is supported by robust pharmacoepidemiological evidence; a large network meta-analysis by Cortese and colleagues, incorporating data from 133 randomized controlled trials, concluded that methylphenidate is the preferred first-choice medication for children and adolescents based on both efficacy and tolerability profiles (24). Furthermore, the ADDUCE study, the largest prospective investigation of methylphenidate safety to date, demonstrated reassuring long-term safety over a 2-year follow-up period in 1,410 children and adolescents, with no significant effects on growth or increased risk of psychiatric adverse events compared to untreated ADHD patients (25, 26). Students also demonstrated awareness of common methylphenidate side effects, with insomnia (78.3%), headache (77.3%), and decreased appetite (71.9%) being most frequently identified, which aligns with established side effect profiles documented in these large-scale studies (24, 25, 27).

Several limitations of this study should be acknowledged. First, the cross-sectional design precludes determination of causal relationships and does not allow for assessment of symptom stability over time. Second, the study was conducted at a single institution, which may limit generalizability to other pharmacy programs or regions within Saudi Arabia. Furthermore, the response rate was 18.2%, which may have introduced non-response bias if students who chose to participate differed systematically from those who did not. Third, the ASRS version 1.1 Screener is a self-report instrument designed for screening purposes rather than definitive diagnosis; positive screening results indicate the need for comprehensive clinical evaluation rather than confirmed ADHD. Fourth, the reliance on self-reported data introduces potential response biases, including social desirability bias and recall limitations. Fifth, comorbid psychiatric conditions that may mimic or exacerbate ADHD symptoms, such as anxiety and depression, were not assessed, potentially inflating estimates of positive ADHD symptom screening. Finally, the relatively modest sample size and the reliance on a convenience sample of available students may have limited statistical power to detect smaller associations. In addition, several unmeasured confounders such as anxiety, burnout, and sleep deprivation, which are common in high-pressure academic programs, may have influenced the findings. The possibility of type II error and self-report bias should also be acknowledged, and the survey did not capture whether students were currently taking ADHD medication beyond those with a prior diagnosis.

Future research should address these limitations through multi-institutional studies with larger, more diverse samples. Longitudinal investigations tracking ADHD symptoms and their impact on academic and professional outcomes would provide valuable insights into the natural history of ADHD in pharmacy students (22, 25, 28). Studies incorporating clinical diagnostic interviews alongside screening instruments would help validate estimates of positive ADHD symptom screening and characterize students who screen positive but remain without formal evaluation. Additionally, research exploring barriers to diagnosis and treatment-seeking behaviors in Saudi Arabian healthcare students could inform targeted intervention strategies (23, 24). Investigation of the effectiveness of academic accommodations and support services for students with ADHD in pharmacy education settings represents another important area for future inquiry.

## 5 Conclusion

This study observed a difference between the high proportion of positive ADHD symptom screening (31%) and self-reported prior diagnosis (5.9%) among pharmacy students. These findings support further evaluation of ADHD awareness, referral pathways, and student mental health support services within pharmacy education. In addition, integrating structured educational content on ADHD—including its diagnosis, pharmacotherapy, and patient counseling—may support educational preparedness for managing ADHD in future practice. Furthermore, increasing awareness that ADHD may affect students themselves could facilitate early recognition and appropriate support-seeking behavior.

## Data Availability

The raw data supporting the conclusions of this article will be made available by the authors, without undue reservation.
