# Supplementary material for: Self-reported ADHD symptoms among pharmacy students: prevalence of positive ADHD symptom screening, academic correlates, and implications for educational support
Source: Front Psychiatry. 2026 Jul 23;17:1872812. doi: 10.3389/fpsyt.2026.1872812 (PMC13444369; doi:10.3389/fpsyt.2026.1872812)
Supplement: Supplementary file 1 [file SupplementaryFile1.pdf]

Study Questionnaire

Title: Self-Reported ADHD Symptoms Among Pharmacy Students: Prevalence, Academic Correlates, and Implications for Educational Support

Participant Information:

- You are invited to participate in a research study that aims to estimate the prevalence of self-reported symptoms consistent with ADHD among pharmacy students at Umm Al-Qura University, examine associated demographic and academic factors, and assess students' knowledge of ADHD pharmacotherapy.
- Participation in this study is entirely voluntary. The questionnaire is intended for undergraduate Doctor of Pharmacy (Pharm.D) students across all academic years (1st –6th year) at the College of Pharmacy, Umm Al-Qura University, Makkah, Saudi Arabia and will take approximately 3–4 minutes to complete.
- All responses are anonymous and confidential. No personally identifiable information will be collected, and the data will be used solely for research purposes. Participants may discontinue the survey at any time before submitting their responses without any consequences.
- This study has been reviewed and approved by the Institutional Review Board (IRB) of Umm Al-Qura University (Approval No. HAPO-02-K-012-2025-02-2536).
- If you have any questions regarding this study, please contact the principal investigator at smwali@uqu.edu.sa

Consent to Participate:

By selecting **"Yes"** and proceeding to the questionnaire, you confirm that:

- You are currently a pharmacy student in Saudi Arabia.
- You have read and understood the information provided above.
- You voluntarily agree to participate in this study.

Do you agree to participate in this study?

○ Yes, I agree to participate.

○ No, I do not agree to participate.

1. **What is your age? ***

- ◯ 18-22
- ◯ 23-27
- ◯ 28-32
- ◯ 33+

2. **What is your gender? ***

◯
Male ◯
Female

3. **What year are you in your pharmacy studies? ***

◯ 1st
◯
2nd ◯
3rd ◯
4th ◯
5th ◯
6th

4. What is your current GPA (Grade Point Average) on a scale of 0 to 4.0 *

◯ 0.0 - 1.0

◯ 1.1 - 2.0

◯ 2.1 - 3.0

◯ 3.1 - 4.

5. **How often do you struggle to finalize the last details of tasks (homework, group presentations, or daily tasks), once the challenging parts have been completed?** *

◯

Never

◯

Rarely

◯

Sometime

s ◯

Often

◯ Very often

6. **How often do you have difficulty getting things in order when you have to do a task that requires organization?** *

◯

Never

◯

Rarely

◯

Sometime

s ◯

Often

○ Very often

7. **How often do you have problems remembering appointments or obligations? ***

○

Never

○

Rarely

○

Sometime

s ○

Often

○ Very often

8. **When you have a task that requires a lot of thought, how often do you avoid or delay getting started? ***

○

Never

○

Rarely

○

Sometime

s ○

Often

○ Very often

9. **How often do you move around or wiggle your hands or feet when you have to sit down for a long time? ***

○

Never

○

Rarely

○

Sometime

s ○

Often

○ Very often

10. **How often do you feel overly active and compelled to do things, like you were driven by a motor?** *

○

Never

○

Rarely

○

Sometime

s ○

Often

○ Very often

11. On average, how many hours do you spend studying each day? *

○ Less than 1

hour ○ 1 - 2

hours

○ 3 - 4 hours

○ 5 - 6 hours

○ More than 6 hours

12. **Have you ever been diagnosed with ADHD?***

- ◯ Yes
- ◯ No

13. Are you currently taking medication for ADHD? *

- ◯ Yes
- ◯ No

14. Do you believe you have ADHD? *

- ◯ Yes
- ◯ No

---

15. Which of the following is considered the first-line treatment for ADHD? *

- ◯ Donepezil (Aricept)
- ◯ Carbamazepine (Tegretol)
- ◯ Methylphenidate (Ritalin, Concerta)
- ◯ Levodopa/Carbidopa (Sinemet)

16. Which of the following are known side effects of methylphenidate? (Select three options) *

Please select 3 options.

☐ Insomnia

☐ Headache

☐ Decreased appetite

☐ Urinary retention

☐ Dry mouth
